# Supplementary material for: TNFα blockade overcomes resistance to anti-PD-1 in experimental melanoma
Source: Nat Commun. 2017 Dec 22;8:2256. doi: 10.1038/s41467-017-02358-7 (PMC5741628; doi:10.1038/s41467-017-02358-7)
Supplement: Supplementary file 1 — Supplementary Information [file 41467_2017_2358_MOESM1_ESM.pdf]

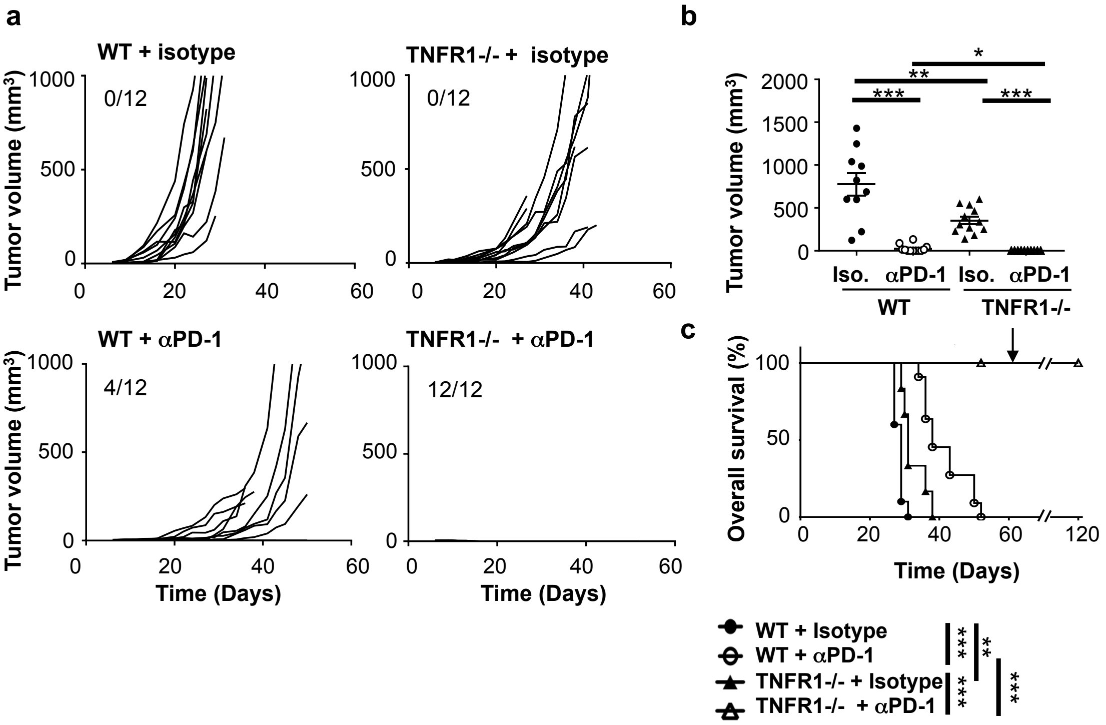

Supplementary Figure 1: TNFR1 deficiency strongly enhances the anti-PD-1 response in experimental melanoma. C57BL/6 wild-type (WT) and TNFR1-deficient mice were intradermally and bilaterally grafted with $3x10^5$ B16K1 melanoma cells prior to intraperitoneal injection of anti-PD-1 antibodies (αPD-1, 10 mg kg^{-1}) or a relevant isotype control (Iso, 10 mg kg^{-1}) at days 6, 9 and 13 (n=6 mice per group). **a,** Tumor volumes were determined with a calliper. Individual curves are depicted for each tumor. Numbers indicate how many tumors regressed out of total tumors. Results are representative of two experiments. **b,** Values determined at day 27 for individual tumors are depicted. Bars represent mean values ± s.e.m and are representative of two experiments. A Mann-Whitney U test was used and differences were considered to be statistically significant when $p<0.05$ (*$p<0.05$; **$p<0.01$; ***$p<0.001$). **c,** Cumulative survival curves. At day 60, surviving mice were challenged with a second injection of B16K1 cells as indicated by the arrow. For statistical analysis of survival, the log-rank test was used and differences were considered to be statistically significant when $p<0.05$ (**$p<0.01$; ***$p<0.001$).

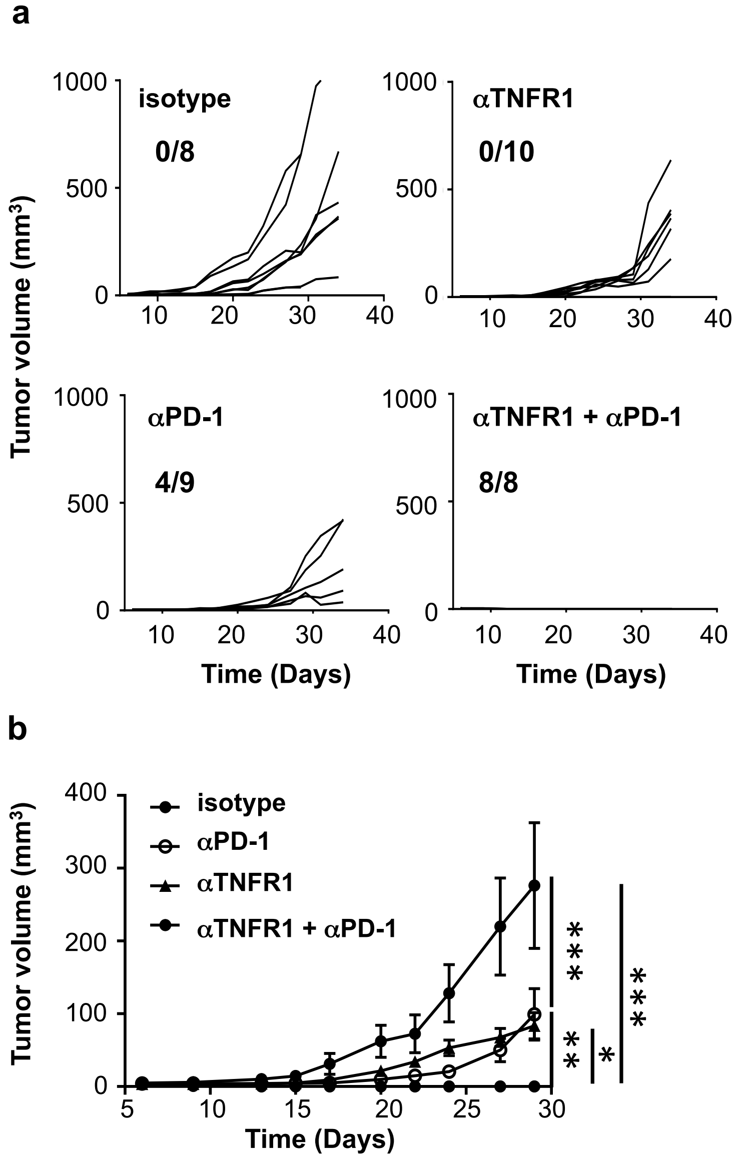

Supplementary Figure 2: anti-TNFR1 blocking antibodies potentiate anti-PD-1 efficacy. C57BL/6 WT mice were intradermally and bilaterally grafted with $3x10^5$ B16K1 melanoma cells. Mice received two injections of anti-TNFR1 and anti-PD-1 antibodies at days 6 and 9 (10 mg kg^{-1}) alone or in combination. Alternatively, mice were injected with isotype control. Tumor volumes were determined with a calliper at the indicated days. **a,** Individual curves are depicted for each tumor. Numbers indicate how many tumors regressed out of total tumors. **b,** Data are means ± s.e.m. of at least 4 mice per group of one experiment. A two-way Anova with Tukey multiple comparison test was used and differences were statistically significant (*p<0.05; **p<0.01; *** p<0.001) at the indicated times.

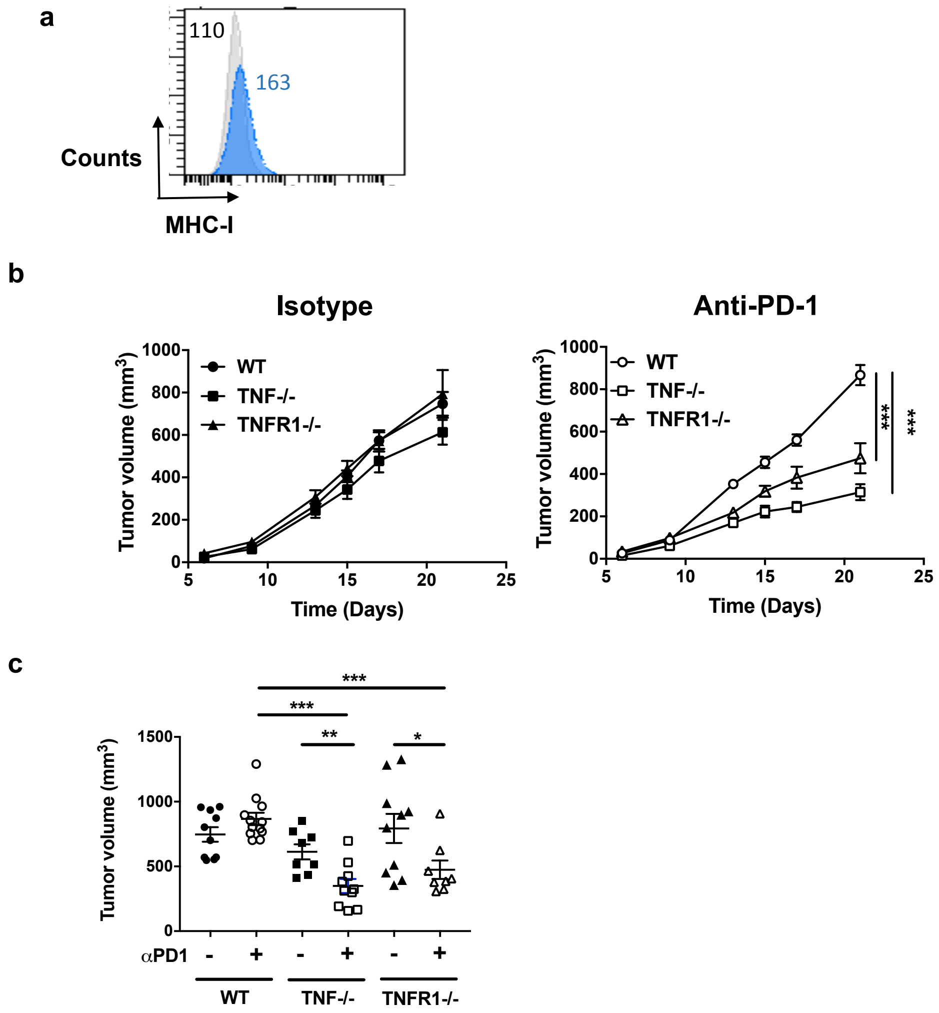

Supplementary Figure 3: Deficiency in TNF or TNFR1 potentiates anti-PD-1 therapy in established Lewis lung carcinoma (LLC) tumors. a, MHC-I expression level on LLC was evaluated using flow cytometry: blue histogram, MHC-I staining; grey histogram, isotype control. Numbers indicate the mean fluorescence intensity. C57BL/6 wild-type (WT), TNF-deficient (TNF-/-) and TNFR1-deficient (TNFR1-/-) mice were intradermally and bilaterally grafted with 4×10^5 LLC cells prior to intraperitoneal injection of anti-PD-1 antibodies (αPD-1, 10 mg kg^{-1}) or a relevant isotype control (Ctrl, 10 mg kg^{-1}) at days 6, 9 and 13. **b,** Tumor volumes were determined with a calliper at the indicated days. Data are mean ± s.e.m of at least 4 mice per group analysed in one experiment. A two-way Anova with Tukey multiple comparison test was used and differences were statistically significant (*p<0.05; **p<0.01; *** p<0.001) at day 21. **c,** Values determined at day 21 for individual tumors are depicted. Bars represent mean values ± s.e.m. A Mann-Whitney U test was used and differences were considered to be statistically significant when p<0.05 (*p<0.05; **p<0.01; ***p<0.001) (b, c).

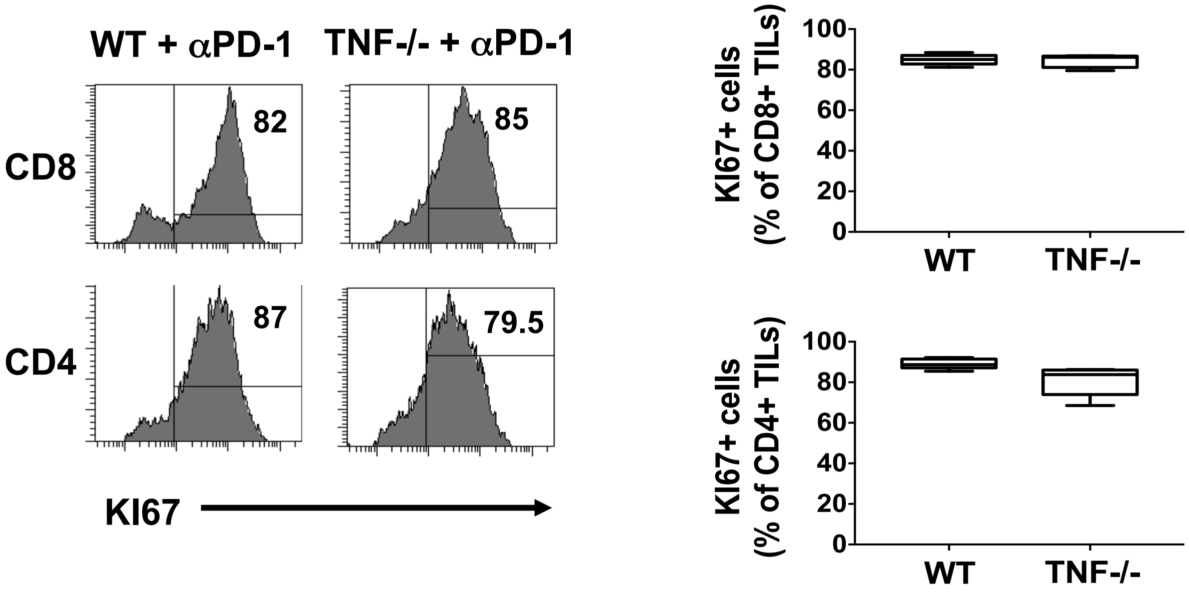

Supplementary Figure 4: TNF deficiency did not modify cell proliferation in CD4+ and CD8+ TIL upon anti-PD-1 therapy. C57BL/6 wild-type (WT) and TNF-deficient (TNF-/-) mice were injected as described in the legend to Fig. 2. At day 10, the TIL content was analysed by flow cytometry. The proportion of CD8+ and CD4+ TILs expressing KI67 was evaluated. Left panels: representative stainings. Right panels: values from one experiment measured in 6 tumors per group are represented as Tukey boxes. No significant differences were found between both groups when using a Mann-Whitney U test.

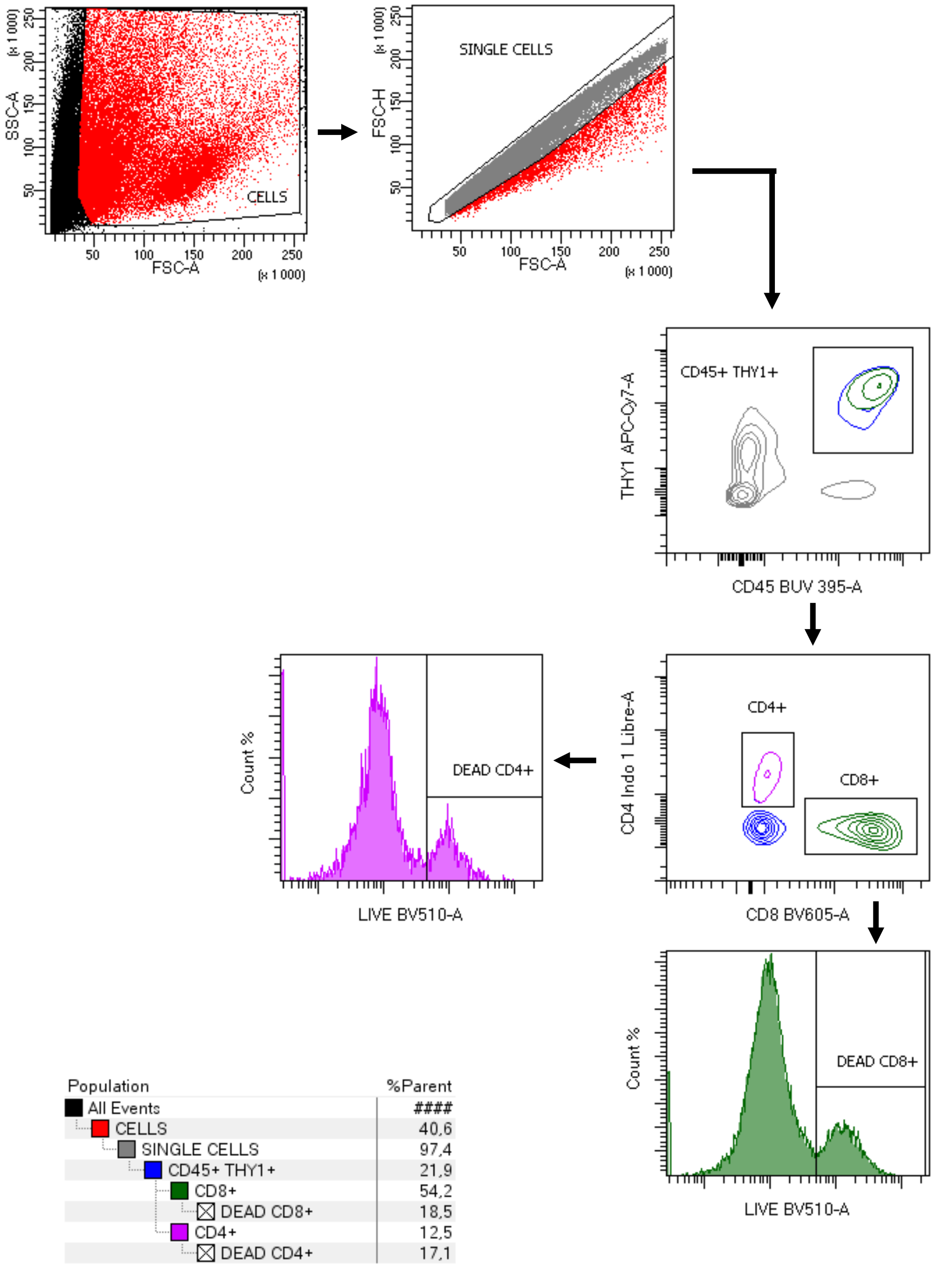

Supplementary Figure 5: Analysis of cell death in TILs. Mice were intradermally and bilaterally grafted with $1x10^6$ B16K1 melanoma cells. At day 10, tumors were collected and dissociated, and tumor cell content was analysed by using flow cytometry after cell incubation with Live/dead reagents, anti-CD45, anti-Thy1, anti-CD4 and anti-CD8 antibodies. The gating strategy of TIL was as follows: (i) cell debris were first excluded from the analysis by using FSC-A and SSC-A parameters; (ii) analysis was next restricted to single cells by using FSC-A and FSC-H parameters; (iii) CD4+ TILs and CD8+ TILs were next gated among CD45+Thy+ cells; (iv) Live/dead reagent staining was analysed as indicated to determine the proportion of dead cells among CD4+ and CD8+ TILs.

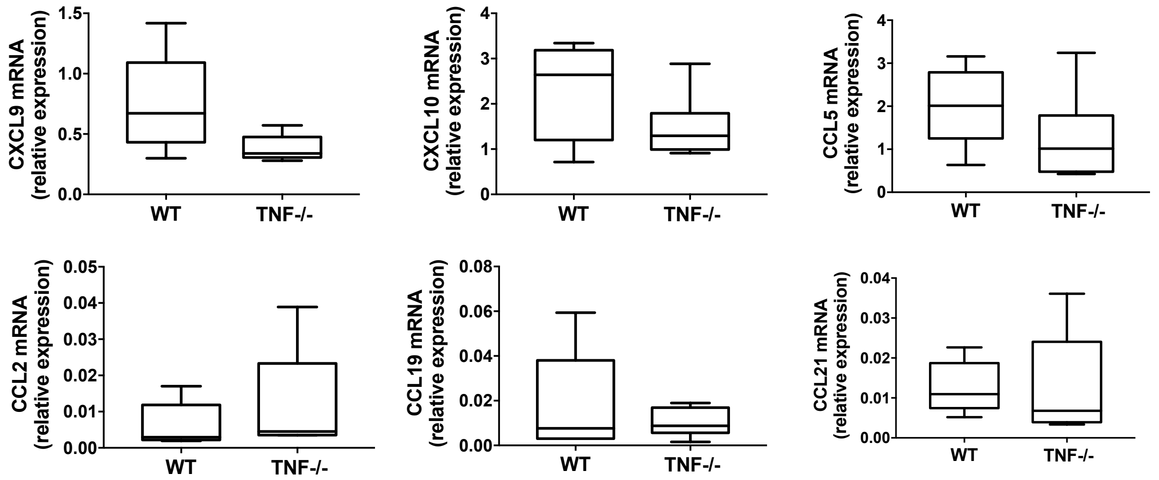

Supplementary Figure 6: Chemokine mRNA expression analysis. C57BL/6 wild-type (WT) and TNF-deficient (TNF-/-) mice were intradermally and bilaterally grafted with $1x10^6$ B16K1 melanoma cells prior to intraperitoneal injection of anti-PD-1 antibodies (αPD-1, 10 mg kg^{-1}) at day 7. Chemokine transcript levels were quantified in tumors from 6 mice per group and relative expression was normalized to housekeeping gene. Data from one experiment are represented as Tukey box. No significant differences were found between both groups by using a Mann-Whitney U test.

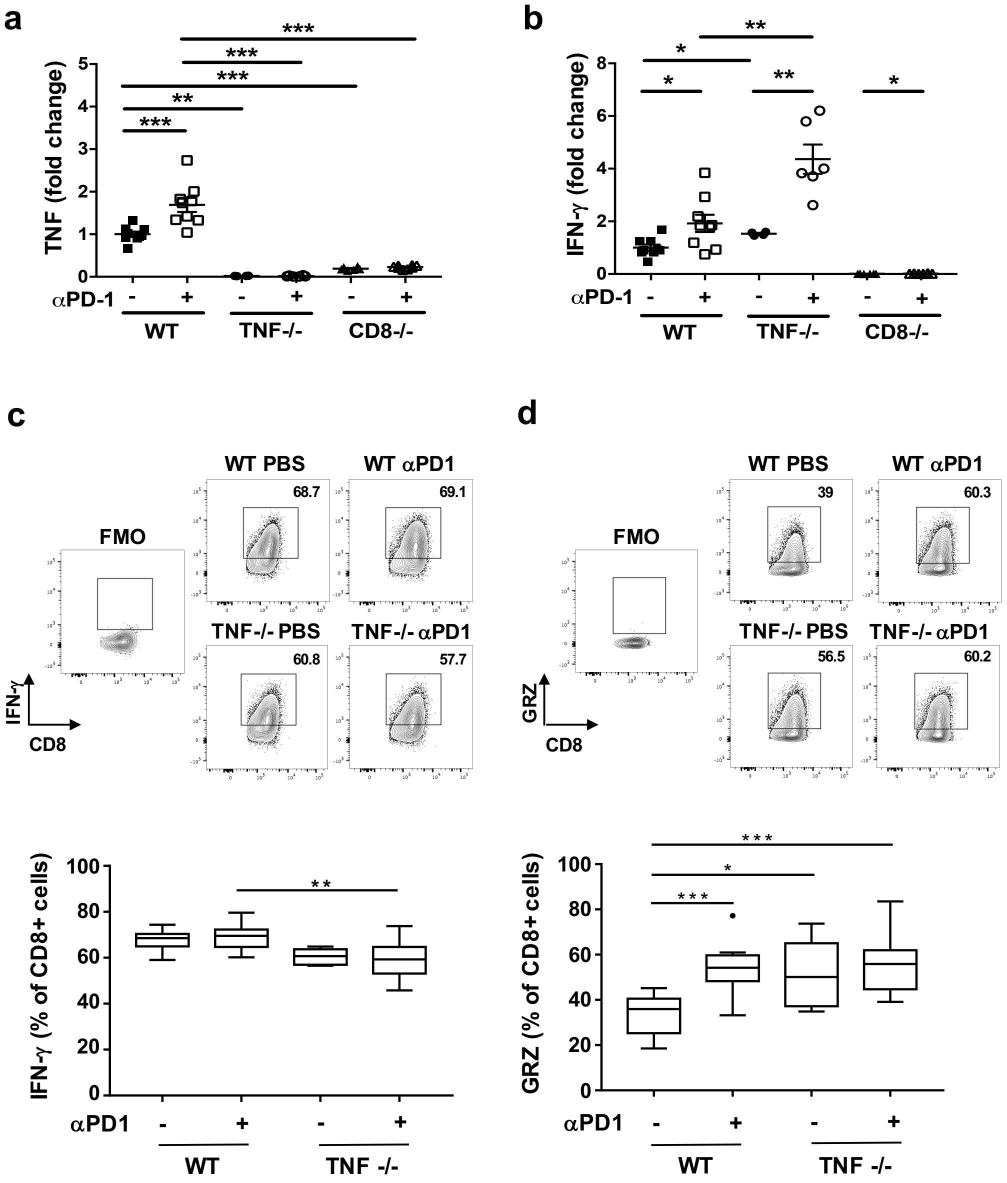

Supplementary Figure 7: IFN-γ and granzyme B production in wild-type and TNF-deficient mice. C57BL/6 wild-type (WT), TNF-deficient (TNF-/-) and CD8-deficient (CD8-/-) mice were injected as described in the legend to Fig. 2. **a and b**, At day 10, TNF (a) and IFN-γ (b) transcript levels were quantified in tumors. Data are means ± s.e.m. of at least 4 tumors per group analysed in one experiment. (Mann-Whitney U test: *$p<0.05$; **$p<0.01$; ***$p<0.001$). **c and d,** The TIL content was analysed by flow cytometry after 4 hours incubation with PMA and ionomycin. The proportion of CD8+ TILs expressing IFN-γ (c) and that of CD8+ TILs expressing granzyme B (GRZ) (d) were determined. FMO: Fluorescence Minus One controls. Upper panels: representative stainings. Lower panels: values measured in 5-12 tumors per group from two experiments are represented as Tukey boxes. (Student's t test: *$p<0.05$; **$p<0.01$; ***$p<0.001$).

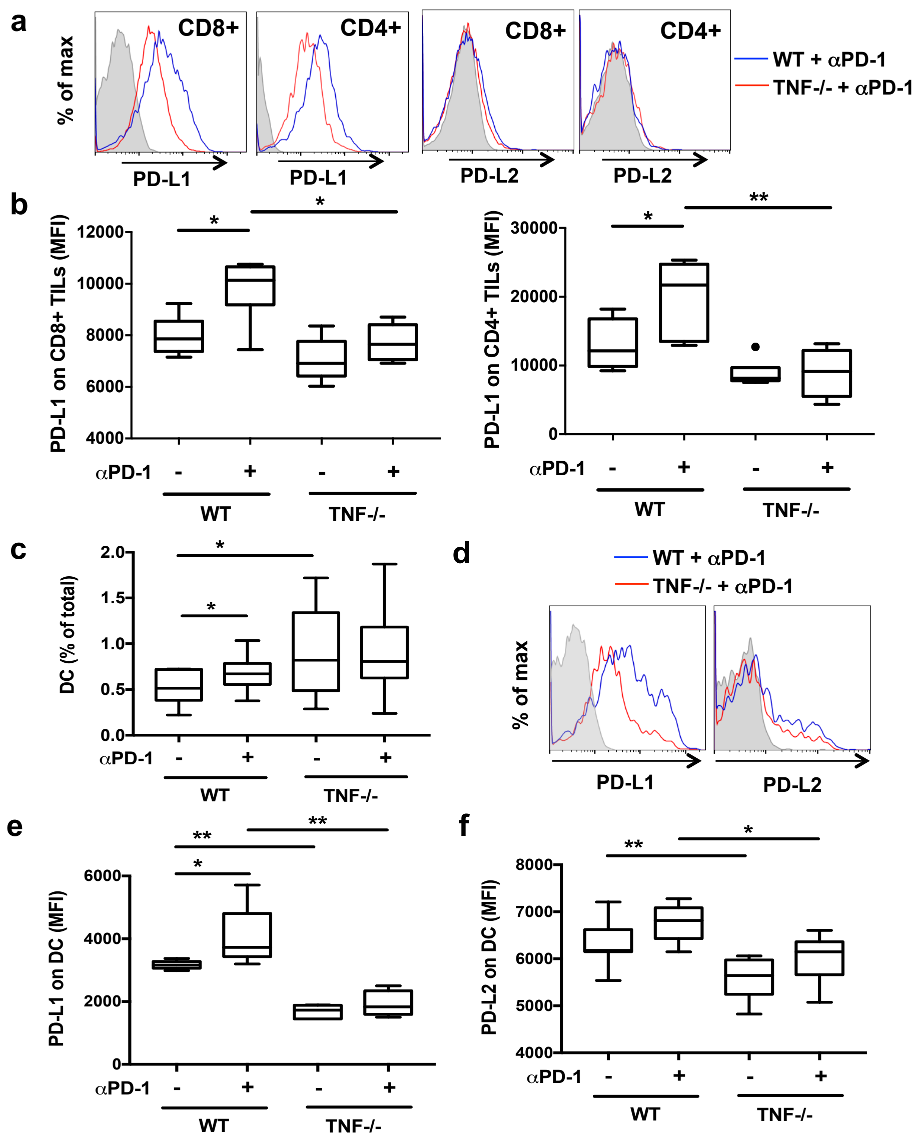

Supplementary Figure 8: TNF induces PD-L1 and/or PD-L2 expression on TILs and DC. WT and TNF-deficient (TNF-/-) mice were injected as described in the legend to Fig. 2. TILs were analysed by flow cytometry on tumors developed at day 10. **a,** Representative staining for PD-L1 (left panels) and PD-L2 (right panels) on CD8+ and CD4+ TILs from WT and TNF-deficient mice injected with anti-PD-1. Grey histograms: isotype control. **b,** Mean of fluorescence intensity (MFI) for the PD-L1 staining on CD8+ TILs (left panel) and CD4+ TILs (right panel). **c,** Percentage of tumor DCs among total cells. **d-f,** Representative plots showing the expression of PD-L1 (left panel) and PD-L2 (right panel) on DCs in tumors from WT and TNF-deficient mice (d). Mean of fluorescence intensity (MFI) of PD-L1 (e) or PD-L2 (f) staining on DCs. **b, c, e and f,** Values measured in at least 6 tumors per group from one (out of two) representative experiment are represented as Tukey boxes (Mann-Whitney U test: *p<0.05; **p<0.01).

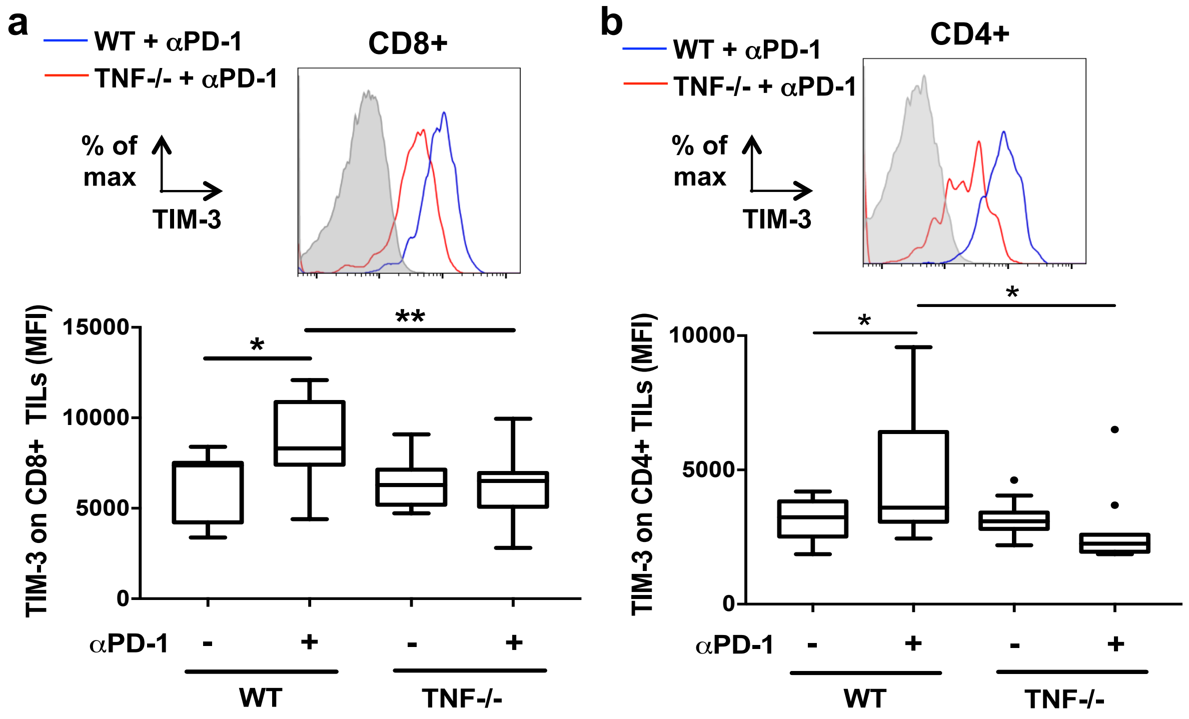

Supplementary Figure 9: TNF deficiency impairs anti-PD-1-treated TIM-3 up-regulation on T lymphocytes. a and b, WT and TNF-deficient (TNF-/-) mice were injected as described in the legend to Fig. 2. TIM-3 expression on CD8+ (a) and CD4+ TILs (b) was determined by flow cytometry on tumors developed at day 10. Upper panels: representative staining on TILs from WT (blue histogram) and TNF-deficient (red histogram) mice injected with anti-PD-1. Lower panels: Mean Fluorescence Intensity (MFI) measured in at least 11 tumors per group from two independent experiments are represented as Tukey boxes (Mann-Whitney U test: *p<0.05; **p<0.01).

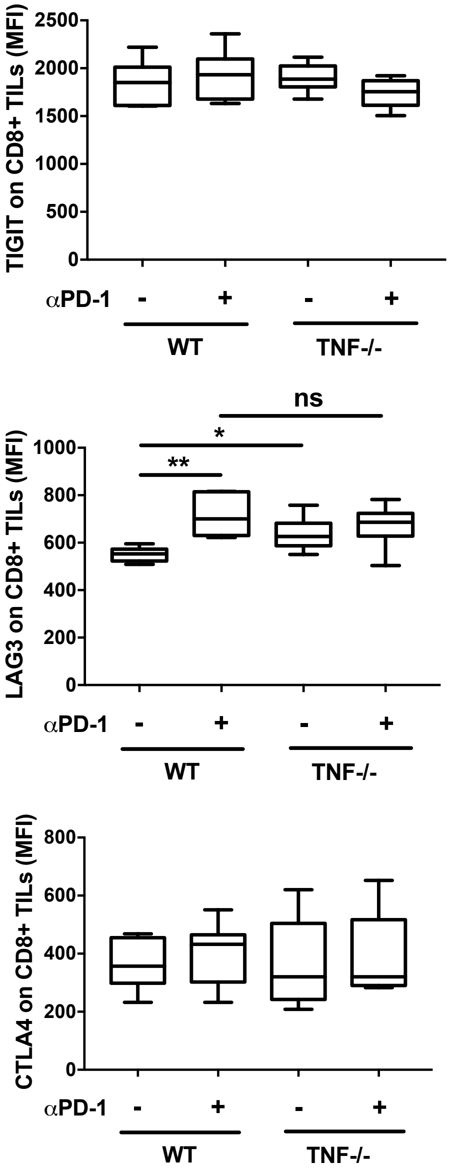

Supplementary Figure 10: TNF does not induce TIGIT, LAG-3 and CTLA-4 expression on CD8+ TILs. The mean of fluorescence intensity (MFI) of TIGIT, LAG3 and CTLA-4 staining on CD8+ TILs was determined by flow cytometry on tumors developed at day 10 in WT and TNF-deficient (TNF-/-) mice that had received one injection of anti-PD-1 antibodies (αPD-1, 10 mg kg^{-1}) or vehicle (PBS) at day 7. Values measured in at least 11 tumors per group from two independent experiments are represented as Tukey box. A Mann-Whitney U test was used (ns: not significant; *$p<0.05$; **$p<0.01$).

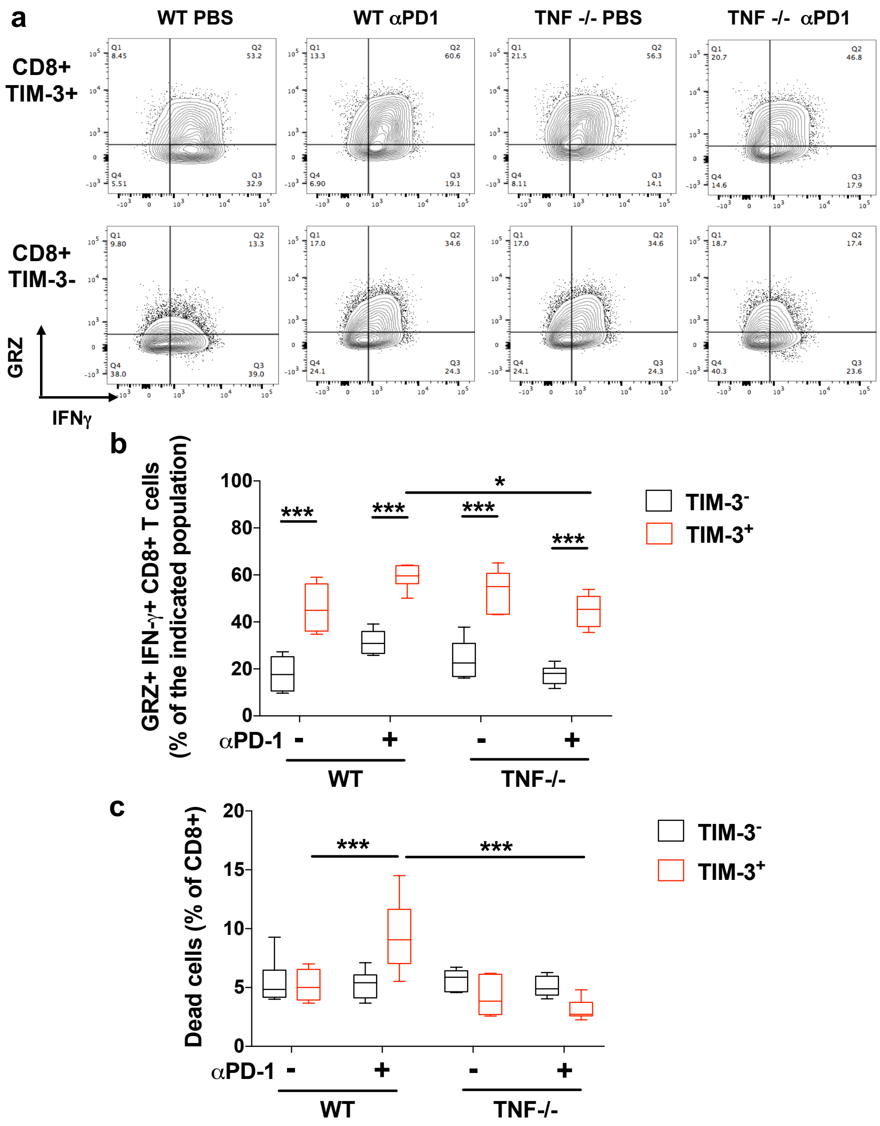

Supplementary Figure 11: IFN-γ and Granzyme B expression in TIM-3+ and TIM-3- CD8+ TILs. WT and TNF-deficient (TNF-/-) mice were injected as described in the legend to Fig. 2. TILs from tumors developed at day 10 were stimulated with PMA/ionomycin in the presence of brefeldin A and the proportion of CD8+ TILs expressing granzyme B (GRZ) and IFN-γ was analysed by flow cytometry. **a,** Representative stainings; values indicate the proportion of cells in the different quadrants. **b,** Quantification of the proportion of GRZ+ and IFN-γ+ cells among the TIM-3+ and TIM-3- CD8+ TILs. **c**, Proportion of cell death of the indicated populations among CD8+ T cells. Values measured in 5-6 mice per group from one experiment are represented as Tukey boxes (b and c: Two-way Anova with Tukey's multiple comparison test: *p<0.05; ***p<0.001).

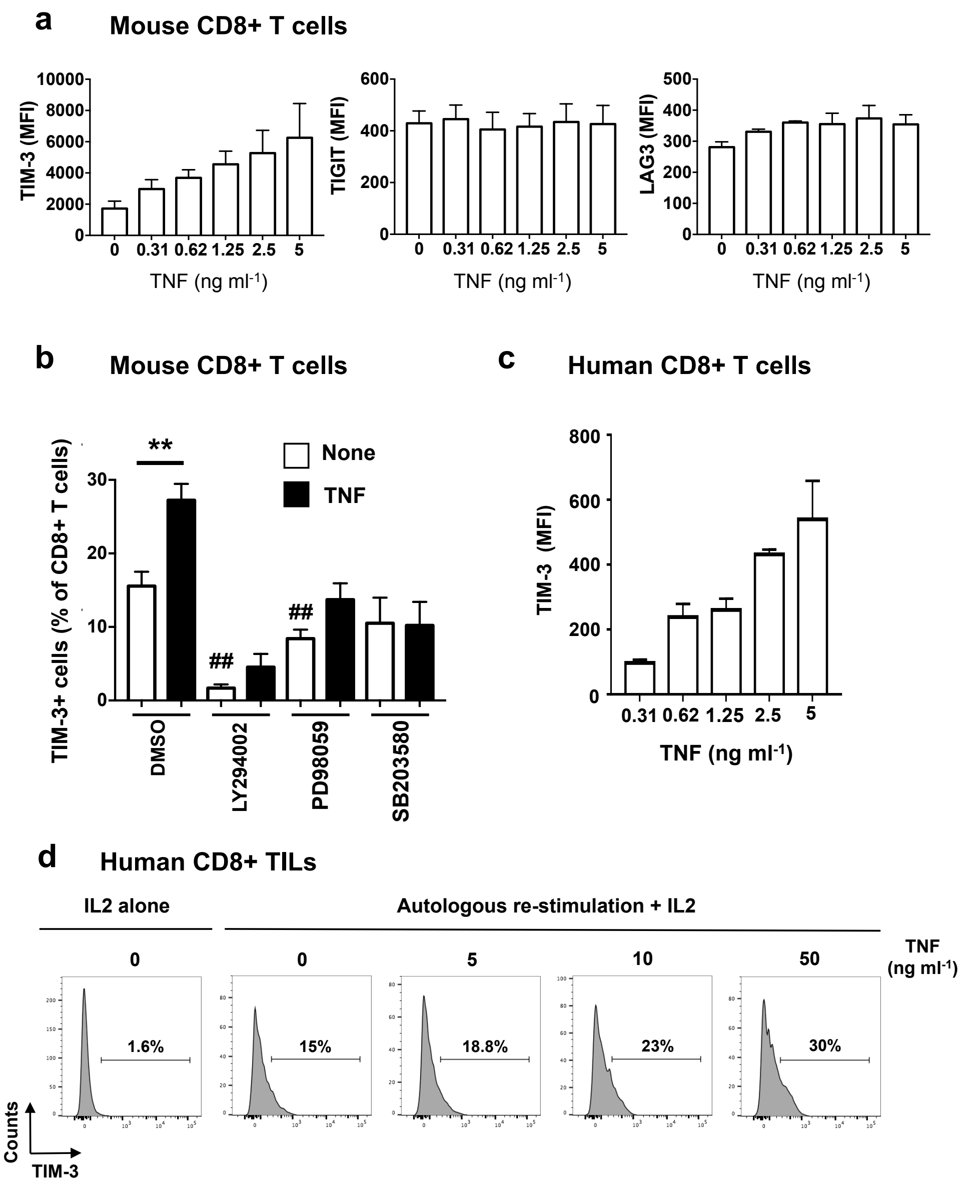

Supplementary Figure 12: TNF dose-dependently induces TIM-3 up-regulation on activated CD8+ T lymphocytes *in vitro*. **a,** Activated CD8+ T cells from WT mice were incubated with murine TNF for 2 days. The mean fluorescence intensity (MFI) of TIM-3, LAG3 and TIGIT was next analysed by flow cytometry. Data are means ± s.e.m. of three independent experiments. **b,** Similar experiment as described in "a" was conducted by incubating cells with 1 µM LY294002 (PI3K inhibitor), 10 µM PD98059 (MEK inhibitor), 1 µM SB203580 (p38 MAPK inhibitor) or vehicle (DMSO) in the presence or absence of 2.5 ng mL^{-1} TNF. Percentages of CD8+TIM-3+ T cells were next determined by flow cytometry. Data are means ± s.e.m. of four independent experiments. A Mann-Whitney U test was used and differences were statistically significant (**$p<0.01$, TNF *vs* vehicle; ##$p<0.01$, LY294002 or PD98059 *vs* vehicle). **c,** Human CD8+ T cells were incubated with different concentrations of human TNF for 2 days. TIM-3 expression was next analysed by flow cytometry. Histograms represent the MFI after subtracting basal fluorescence intensity. Data are means ± s.e.m. of two experiments. **d,** TILs from one human melanoma metastasis (patient 2) were cultured as described in Figs 4b and c with increasing concentrations of human recombinant TNF for 48h. TIM-3 expression on re-stimulated CD8+ TILs was analysed by flow cytometry.

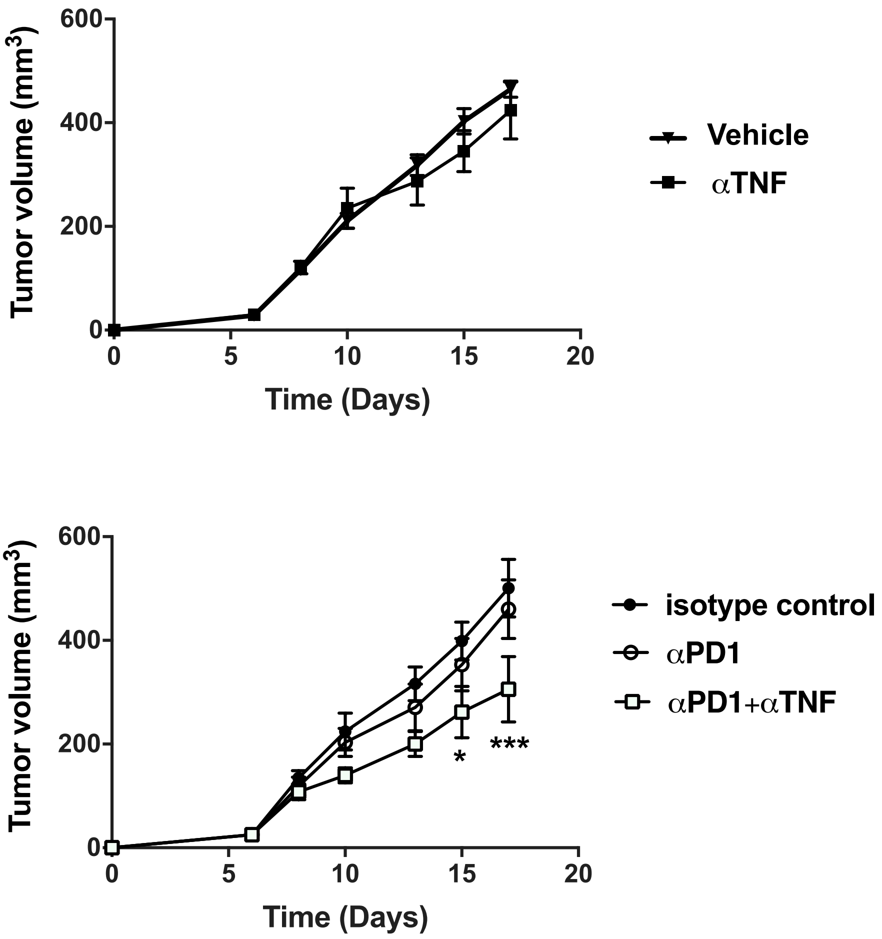

Supplementary Figure 13: anti-TNF antibodies overcome the resistance to anti-PD-1 therapy in experimental breast carcinoma. $1x10^5$ 4T1 breast carcinoma cells were orthotopically injected into the mammary fat pad of WT Balb/c mice. Mice were next injected with vehicle, isotype control, anti-TNF, anti-PD-1 or the combination of anti-PD-1 and anti-TNF (10 mg kg^{-1} of each antibody). A two-way Anova with Tukey multiple comparison test was used and differences were statistically significant (*$p<0.05$; *** $p<0.001$) at the indicated times between the group of mice, which received the combo anti-PD-1 and anti-TNF and the isotype control group. Data are mean values ± s.e.m. of 6 mice per group from one experiment.
